# Febrile Seizures: Etiology, Prevalence, and Geographical Variation

**Published:** 2014

**Authors:** Ali DELPISHEH, Yousef VEISANI, Kourosh SAYEHMIRI, Afshin FAYYAZI

**Affiliations:** 1 Prevention of Psychosocial Injuries Research Center, Ilam University of Medical Sciences, Ilam, Iran; 2 Department of Clinical Epidemiology, Ilam University of Medical Sciences, Ilam, Iran; 3 The Student Research Committee, Ilam University of Medical Sciences, Ilam, Iran; 4 Department of Community Medicine, Ilam University of Medical Sciences, Ilam, Iran; 5 Department of Pediatric, Neurology, Hamaden University of Medical Sciences, Hamadan, Iran

**Keywords:** Febrile seizure, Iran, Meta–analysis, Pediatrics

## Abstract

**Objective:**

Febrile seizures (FSs) are the most common neurological disorder observed in the pediatric age group. The present study provides information about epidemiological and clinical characteristics as well as risk factors associated with FS among Iranian children.

**Materials & Methods:**

On the computerized literature valid databases, the FS prevalence and 95% confidence intervals were calculated using a random effects model. A metaregression analysis was introduced to explore heterogeneity between studies.

Data manipulation and statistical analyses were performed using Stata10.

**Results:**

The important viral or bacterial infection causes of FSs were; recent upper respiratory infection 42.3% (95% CI: 37.2%–47.4%), gastroenteritis21.5% (95% CI: 13.6%–29.4%), and otitis media nfections15.2% (95% CI: 9.8%- 20.7%) respectively. The pooled prevalence rate of FS among other childhood convulsions was 47.9% (95% CI: 38.8–59.9%). The meta–regression analysis showed that the sample size does not significantly affect heterogeneity for the factor ‘prevalence FS’.

**Conclusion:**

Almost half of all childhood convulsions among Iranian children are associated with Febrile seizure.

## Introduction

Febrile seizure (FS) is the most common neurological disorder observed in the pediatric age group. It has been reported that one in every 25 children in the population will experience at least one FS during their childhood (1). The International League against Epilepsy (ILAE) has defined FS as seizure events in infancy or childhood are featured with temperatures over 38°C without any evidence of acute electrolyte imbalances in CNS infection or history. A child with FS often loses consciousness, shakes, and moves limbs on both sides of the body. Most FSs occur during the first day of a child’s fever (2).

The direct cause of FS is unknown, but the most important associated factors are fever, epilepsy, hypoglycemia, hypocalcaemia, head injury, poisoning and drug overuse, respiratory infection, or gastroenteritis (3–5). The association between seizure and bacterial infection is conventional (6, 7). Although, FS may cause great fear and concern for parents, it usually does not produce lasting effects (8). The types of FS are also important.

Children who have focal or lateralized FS, prolonged (particularly lasting more than an hour) or seizures that affect only a part of the body, or that recur within 24 hours, are more hazardous (9).

Many studies have already revealed etiology, prevalence, and geographical variation of apparent FSs among Iranian children across the country (10–13). Even though, there are few literature review articles are available looking at the prevalence, etiology, and geographical variation in children with an apparent FS from Iran. However, we used only papers with precise methodology and noted more recent publications (11-13, 16-32). To do so, we conducted a systematic review and meta–analysis to provide epidemiological characteristics including prevalence, etiology, and geographical variation of the FSs among Iranian children. 

## Methods & Materials

The search strategy, selection of publications, and the reporting of results for the review were conducted in accordance with the PRISMA guidelines. Literatures related to FS characteristics in Iranian children were acquired through searching Scientific Information Databases (SID), Global Medical Article Limberly (Medlib), Iranian Biomedical Journal (Iran Medex), Iranian Journal Database (Magiran) as well as international databases including PubMed/Medline and ISI Web of Knowledge were searched for published data related to FS in Iran. The search strategy was limited to the Persian and/or English languages and articles published until Feb 2012 were considered. All publications with medical subject headings (MeSH) and keywords in the title, abstract, and text for words including febrile seizure were investigated. Iranian scientific databases were searched only using the keyword ‘febrile seizure’, as these databases do not distinguish synonyms from each other and do not allow sensitive search operations using linked terms such as ‘AND’, ‘OR’, or ‘NOT’. 

Consequently, single keywords were searched in inner databases. MeSh keywords including seizures, pediatrics and Iran were assessed combined with the operator “OR” vs “AND” for outer databases. The search string in PubMed was (Seizures [Title]) OR Pediatrics [Title]) AND Iran [Affiliation]) 

**Selection and quality assessment of articles**

All identified papers were critically appraised independently by two independent reviewers.

Disagreements were resolved through discussion. Appraisal was guided by a checklist assessing clarity of aims and research questions. The inclusion criteria were as follows: 1. Studies in the mentioned databases with full text, despite the language of original text; 2. Hospital– based data; 3. Reporting among Iranian children; and 4. Studies with overlapping time and sample collection from the same origin. The following exclusion criteria were also applied: 1. inappropriate design; 2. Inadequate reporting of results, i.e., studies not reporting prevalence data for relevant outcomes.

**Data extraction**

Data were extracted using a standardized and pre–piloted data extraction form. Data extraction was undertaken by the first reviewer and checked by a second reviewer. However, the process was discussed and piloted by both reviewers. All identified papers were critically appraised independently by both reviewers. Disagreements were resolved through discussion. Appraisal was guided by a checklist assessing clarity of aims and research questions. Information was extracted from author, title, year, setting of study, sample selection, sample size, study type, seizure types, age, and prevalence. Therefore, risk of bias for inadequate reporting was reduced. All data– abstraction forms were reviewed and eligible papers were entered into the meta–analysis.

**Statistical analysis**

The random effects model was used for combining results of studies in meta–analysis. Variance for each study was calculated using the binomial distribution formula. The presence of heterogeneity was determined by the Der Simonian–Laird (DL) approach (14).

Significance level was <0.1 and I2 statistic for estimates of inconsistency within the meta–analyses. The I2 statistic estimates the percent of observed between–study variability due to heterogeneity rather than to chance and ranges from 0 to 100% (values of 25%, 50% and 75% were considered representing low, medium, and high heterogeneity, respectively). A value of 0% indicates no observed heterogeneity while100% indicates significant heterogeneity (15). For this review, we determined that I2 values above 75% were indicative of significant heterogeneity warranting analysis with a random effect model as opposed to the fixed effect model to adjust for the observed variability. This heterogeneity was further explored through subgroup analyses and meta– regression. A univariate and multivariate approach were employed to assess the causes of heterogeneity among the selected studies. The Egger test was conducted to examine potential publication bias. Data manipulation and statistical analyses were done using STATA software, version 11.2. P–values <0.05 were considered statistically significant.

## Results

**Fig 1:**
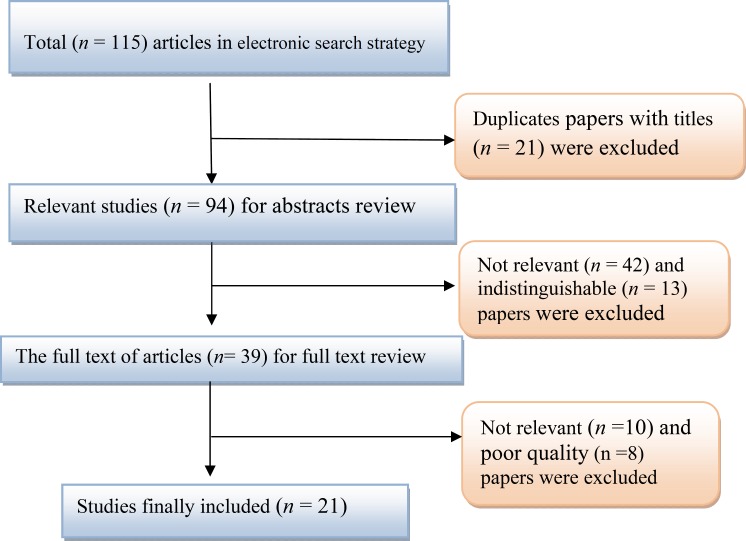
Results of the systematic literature search

Overall, 115 studies (1study in Pub Med, 114 studies in other databases) were identified. Of them, 94 studies were excluded based on the inclusion and exclusion criteria (Figure 1). Finally, 21 articles including one in English (10) and 20in Persian (11–13, 16–32)were adopted (Figure1). On a whole 4599 children with FS including 2734 males and 1865 females included in Meta analysis. Prevalence of FS according to the age of children under 2 years and 2 to 6 years were 55.8% (95% CI: 50.4–61.2%) and 44.1% (95% CI: 38.8–62/2%), respectively (Table1).

**Etiology and prevalence of febrile seizures**

**Fig 2:**
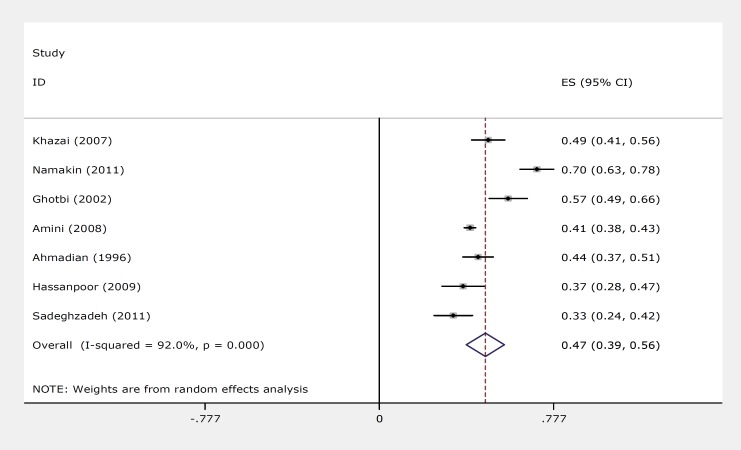
Forest plots of recurrent FS for random effects meta–analyses

The important viral or bacterial infection causes of FSs were recent upper respiratory infection42.3% (95% CI: 37.2%–47.4%), gastroenteritis21.5% (95% CI: 13.6%–29.4%), otitis media infections15.2% (95% CI: 9.8%–20.7%), pneumonia8.7% (95% CI: 5.4%–11.9%), urinary infections3.2% (95% CI: 1.3%–5.0%), rosella 2.0% (95% CI: 0.02%–3.8%), and other infections12.8% (9.8%–15.8%). The pooled prevalence rate of childhood febrile seizure compared to other childhood seizures in Iran was 47.9 %(95% CI 12.3–29.5%) (Figure2).

Prevalence of simple and complex febrile seizure were 69.3% (95% CI: 19.6–31.0) and 28.3% (95% CI: 59.5– 79.0), respectively. Generalized seizures are classified into a number of categories depending on their behavioral effects. Tonic–colonic seizures the prevalence rate among other types of generalized seizures was 78.9% (95%CI: 68.8%–89.2%).

**Table 1:** Feature of childhood febrile seizure at different regions of Iran

Study location (city)	First Author(year)	Study period	No. of patients	Gender (Male) No (%)	Data collection procedure
Yazd	fallah(2008)	2004-2005	139	63(0.55)	Hospital
Yazd	Golestani(2008)	2002-2005	100	59(0.59)	Hospital
Kerman	Hosseininasab(2006)	2000-2002	115	68(0.59)	Hospital
Mashhad	Ashrafzadeh(2002)	2001-2002	50	35(0.70)	Hospital
Zahedan	Khazai(2007)	2005-2006	178	94(0.53)	Hospital
Birjand	Namakin(2011)	2006-2007	145	84(0.61)	Hospital
Bandar Abbas	Moayedi(2001)	2001-2002	181	112(0.62)	Hospital
Sanandaj	Ghotbi(2002)	2000-2001	115	70(0.61)	Hospital
Isfahan	Amini(2008)	2005-2007	1486	892(0.60)	Hospital
Zanjan	Kazemi(2001)	2000-2001	50	33(0.66)	Hospital
Kashan	Talebian(2006)	2001-2002	120	72(0.60)	Hospital
Tehran	Khodapanahande(2001)	2007-2008	107	64(0.60)	Hospital
Tabriz	Barzegar(2006)	2001-2003	582	321(0.55)	Hospital
Bushehr	Sanaidashti(2006)	2005-2006	102	64(0.65)	Hospital
Bandar Abbas	Ahmadian(1996)	1996-1997	211	127(0.60)	Hospital
Tehran	Hassanpoor(2009)	2003-2005	103	64(0.62)	Hospital
Babel	Rasholi(1999)	1999-2000	230	138(0.60)	Hospital
Zanjan	Sadeghzadeh(2011)	2005-2006	117	64(0.55)	Hospital
Tehran	Ehsanypoor(2004)	1997-2007	245	140(0.57)	Hospital
Ilam	Mohammadi(2008)	2007-2008	172	98(0.57)	Hospital
Ahvaz	Dehdashtian(2008)	2003-2008	94	54(0.57)	Hospital

(Squares represent effect estimates of individual studies with their 95% confidence intervals of prevalence FS with size of squares proportional to the weight assigned to the study in the meta–analysis. The diamond represents the overall result and 95% confidence interval of the random–effects meta–analysis). 

**Geographical variation of febrile seizures**

A significant geographic discrepancy on prevalence of FS was also observed in different parts of the country.

Subgroup analysis based on the type of climate showed no interaction with prevalent of FS. Prevalence rate of FS among other childhood convulsions in central Iran was 40.03% (95% CI: 37.09%–42.07%), in the east it was 59.4% (95%CI: 38.2%–80.7%), 44.1% (95% CI: 37.4%–50.8%) in the south, and 57.5% (95% CI: 49.1%–65.9%) in western of Iran. According to the data, the lowest prevalence was observed in north of the country 33.0% (95% CI: 24.5%–41.5%).

**Meta–regression analysis**

Meta–regression, thus, helps explore several possible reasons for the observed heterogeneity among the studies Meta–regression showed an association between year of study and prevalence rate of FS as well as it shows causes of the variability in the results of studies. 

Meta- regression showed variability in prevalence of FS a non significant effect for sample (Reg Coef = 0.017, p= 0.11). Therefore studies with large sample size show prevalence rate of FS high in comparison with studies with small sample size. Meta-regression analysis found that year conducted of the studies significantly affects heterogeneity for the factor ‘prevalence rate FS’ (Reg Coef = .00030, p= 0.026). Publication bias is the term for what occurs whenever the research that appears in the published literature is systematically unrepresentative of the population of completed studies. There was no evidence of publication bias (Egger’s test β0: 0.04; p=0.96) so we tried considered the most of published articles in this subject.

## Discussion

This systematic review aimed to provide epidemiological characteristics of FSs based on 21 separate samples (from 115 publications) based on 4599 neonates. The pooled prevalence of childhood febrile seizures (among other convolutions) in Iran was 47.9% (95% CI; 38.8–59.9%). 

Complex FSs was seen in 28.3% (95% CI: 59.5–79.0) of patients in this study, although other studies have reported a range of prevalence 6.7%–35% (33, 34). This difference in findings may be due to a variety of reasons, including ethnic and geographic differences, better diagnosis of partial seizures and improved methods of patient selection (8). 

According to the national epidemiological survey, the prevalence rate of FSs rate has been decreasing from childhood to adulthood in community trials (35).This is consistent with the findings in the present study, that FS in children under 2-years is higher than for 2- and 6-years of age,58.8% as well as 41.2%, respectively. 

Tonic-colonic seizures the prevalence rate among other types of generalized seizures was 78.9% (95%CI: 68.8%–89.2%).Generalized seizures are classified into a number of categories depending on their behavioral effects. Tonic-colonic seizures are most commonly associated with epilepsy and seizures in general (36, 37). In children between the ages of 6 and 60 months, a simple FS is a benign and common event, and nearly all children have an excellent prognosis. Generalized seizure more associated with susceptibility to epilepsy (38). As epilepsy is most likely due to genetic predisposition rather than structural damage to the brain caused by recurrent simple FSs, there is no evidence that prophylactic treatment of children with simple FS would reduce the risk (39). However, no study has shown that successful treatment of simple FSs can prevent the later development of epilepsy. Further, there is no evidence to date that simple febrile seizures can cause structural damage to the brain (5).

Our study is the first systematic review and meta-analysis that preformed to measure the risk of bacterial infection causes in children with FSs in fewer than for six–year age group. According to the results, urinary infections were 3.2 %( 95% CI: 1.3%–5.0%) in the previous study conducted by Lee et al (1991) and Bauchner (1987).

There were some considerations for bacterial infection playing a role in incidence of FSs, frequency of urinary tract infection was5%, and 1.7%, respectively (6, 7). 

Bacterial meningitis was very uncommon in children diagnosed with FSs inconsistent with our results. Results in this systematic review showed that 8.7% (95% CI: 5.4%–11.9%) were positive, for Streptococcus pneumonia in comparison with Teach et al (1999) whom reported 2.9% (95%CI 0.6–5.2) in children with FSs (40).

Some limitations in the present study need to be addressed. It was an observational study and patients were not randomly selected. Therefore, selection bias and confounding seems to be expected. Meanwhile, the authors’ ability to assess the quality of studies was limited by the fact that many studies failed to offer detailed information of selected subjects or valid data on important factors. Our analysis also suggests the need for large population-based incidence studies of febrile seizure, particularly in children under six year age, to generate more accurate estimates as well as provide a reasonably robust assessment of heterogeneity.
